# Changes in Hydroxyurea Use Among Youths Enrolled in Medicaid With Sickle Cell Anemia After 2014 Revision of Clinical Guidelines

**DOI:** 10.1001/jamanetworkopen.2023.4584

**Published:** 2023-03-24

**Authors:** Sarah L. Reeves, Hannah K. Peng, Jeffrey J. Wing, Lindsay W. Cogan, Alka Goel, David Anders, Nancy S. Green, Lynda D. Lisabeth, Kevin J. Dombkowski

**Affiliations:** 1 Susan B. Meister Child Health Evaluation and Research Center, Department of Pediatrics, University of Michigan, Ann Arbor; 2 Department of Epidemiology, School of Public Health, University of Michigan, Ann Arbor; 3 Division of Epidemiology, College of Public Health, Ohio State University, Columbus; 4 Office of Quality and Patient Safety, New York State Department of Health, Albany; 5 Department of Health Policy, Management and Behavior, School of Public Health, University at Albany, Albany, New York; 6 Department of Pediatrics, Division of Pediatric Hematology, Oncology and Stem Cell Transplantation, Columbia University Medical Center, New York, New York

## Abstract

**Importance:**

Youths with sickle cell anemia (SCA) are at risk of pain crises, stroke, and early death. Complications can be reduced by the oral disease-modifying medication hydroxyurea, and in 2014, the National Heart, Lung, and Blood Institute published revised guidelines that hydroxyurea should be offered to youths aged 9 months and older with SCA regardless of disease severity.

**Objective:**

To describe changes in hydroxyurea use among youths with SCA before and after release of the National Heart, Lung, and Blood Institute guidelines.

**Design, Setting, and Participants:**

This cross-sectional study was conducted using administrative data from 2010 to 2018 from Michigan and New York State (NYS) Medicaid programs. The study population included youths aged 1 to 17 years with SCA enrolled in the Michigan or NYS Medicaid programs for at least 1 year (Michigan: 2010-2018; NYS: 2012-2018). Youths with SCA were identified using validated claims–based definitions. Data were analyzed from June to October 2020.

**Main Outcomes and Measures:**

The main outcome was hydroxyurea use characterized as mean annual counts of days’ supply of filled hydroxyurea prescriptions. Rates of hydroxyurea use over time were assessed using regression models (Michigan: zero-inflated negative binomial; NYS: negative binomial). Models included indicators for periods before and after guideline release.

**Results:**

A total of 4302 youths with SCA (2236 males [52.0%]; 2676 born 2005-2017 [62.2%]; 150 Hispanic [3.5%], 2929 non-Hispanic Black [68.0%], and 389 non-Hispanic White [9.0%]) contributed 12 565 person-years. The mean (SD) annual days’ supply of hydroxyurea was 47.2 (93.6) days per youth in Michigan and 97.4 (137.0) days per youth in NYS. In Michigan, there was an increase in the odds of having nonzero days’ supply after the guidelines were released (odds ratio, 1.52; 95% CI, 1.07-2.14). In NYS, no change was seen in the mean days’ supply of filled hydroxyurea.

**Conclusions and Relevance:**

These findings suggest that hydroxyurea was substantially underused among youths with SCA, despite establishment as the primary disease-modifying therapy for SCA, and that there was incomplete clinician or patient uptake of newly released guidelines. Results suggest that expanding use of hydroxyurea may require a multifaceted approach that includes addressing multiple system- and patient-level barriers.

## Key Points

**Question:**

How did hydroxyurea use change among youths insured by Medicaid with sickle cell anemia (SCA) in Michigan and New York State after the National Heart, Lung, and Blood Institute released revised clinical guidelines in 2014?

**Findings:**

In this cross-sectional study of 4302 youths with SCA, there was an increase in the odds of having nonzero days’ supply of hydroxyurea in Michigan; however, in New York State, no change was seen in mean days’ supply of filled hydroxyurea prescriptions.

**Meaning:**

These findings suggest that increasing hydroxyurea use may require a multifaceted approach that addresses multiple system- and patient-level barriers.

## Teaser

This cross-sectional study investigates changes in the use of hydroxyurea among youths insured by Medicaid with sickle cell anemia after the 2014 revision of clinical guidelines.

## Introduction

Sickle cell disease affects approximately 100 000 individuals in the US, most of whom are from racial and ethnic minoritized groups.^1,2,3,4,5^ Sickle cell disease has many different subtypes, including sickle cell anemia (SCA), defined as genotypes HbSS and HbS-β^0^-thalassemia. SCA is most common and confers the most severe morbidity among subtypes. Acute chest syndrome, infection, stroke, and pain crises are the most common disease symptoms among youths with SCA.^6,7,8,9^ Individuals with pain crises are more likely to experience lower quality of life, more frequent school absences, depression, and impaired peer relationships compared with those without pain crises.^10,11,12^ Pain crises are responsible for most hospitalizations and emergency department visits among youths with SCA.^13^ Given these outcomes, pain has debilitating effects on youths with SCA and is a substantial burden on families and the health care system.^10,11,12^ Youths aged 17 years or younger with SCA had 1.5 to 2.0 emergency department or inpatient stays per person per year from 2005 to 2006.^14^ Pain was the most commonly cited reason for emergency department visits by youths with SCD.^15^

The disease-modifying therapy hydroxyurea can reduce the frequency of pain crises by increasing the amount of fetal hemoglobin in the blood. This decreases the likelihood of sickle hemoglobin polymerization, with subsequent sickling and hemolysis.^16,17^ Among youths with SCA, use of hydroxyurea therapy is associated with lower rates of initial and recurrent pain crises, hand-foot syndrome (dactylitis), acute chest syndrome, overall acute care use, and early mortality compared with no hydroxyurea therapy.^18,19,20,21,22^ Prior to 2014, the National Heart, Lung, and Blood Institute (NHLBI) recommended hydroxyurea therapy for children and adolescents with SCA who had specific indications, such as frequent pain episodes or history of acute chest syndrome.^6^ Given the association of hydroxyurea with reductions in these morbidities, including in very young children enrolled in the Pediatric Hydroxyurea Phase III Clinical Trial (BABY HUG trial),^23^ these recommendations were updated in 2014 to indicate that all youths with SCA starting from age 9 months should be offered hydroxyurea regardless of disease severity.^17^

Despite the association of hydroxyurea therapy with improved clinical and economic outcomes, consistent hydroxyurea use among youths with SCA has been low. At least half of youths who met indication criteria under the previous guidelines (ie, before 2014) did not receive this medication.^18,24,25,26,27^ Among all youths with SCA, fewer than 25% received any hydroxyurea within the year prior to release of the updated guidelines.^18,24,25,26,27^ More recent studies after the updated NHLBI guidelines have shown slightly increased hydroxyurea use, but the extent to which these guidelines are associated with these rates has not been assessed to our knowledge.^28,29,30,31^ Therefore, the objective of this study was to describe changes in hydroxyurea use among youths with SCA from before to after release of the 2014 NHLBI guidelines in 2 states: Michigan and New York. We hypothesized that hydroxyurea use would increase over time across both states after release of the guidelines.

## Methods

This cross-sectional study was approved by the University of Michigan institutional review board (IRB). A waiver of informed consent was granted by the University of Michigan IRB per 45 CFR §46.116. We followed the Strengthening the Reporting of Observational Studies in Epidemiology (STROBE) reporting guideline for cross-sectional studies.

We conducted a serial cross-sectional study using administrative data from Michigan Medicaid (2010-2018) and New York State (NYS) Medicaid (2012-2018), including enrollment history, demographics, and claims for services furnished; data were acquired and analyzed from June to October 2020. Race and ethnicity data were collected through administrative claims databases. Options for race and ethnicity varied by state and year and so were collapsed based on Pew Report guidelines^32^ into the following categories: American Indian or Alaska Native, non-Hispanic; Asian or Pacific Islander, non-Hispanic; Black, non-Hispanic; Hispanic, regardless of race; White, non-Hispanic; and unknown, non-Hispanic. In some years, race and ethnicity were combined at the administrative database level. In other years, they were not, so these variables were collapsed into Pew Report categories. Race and ethnicity were assessed to describe the demographics of the study population. These states are home to many youths with SCA, and most youths with SCA are enrolled in Medicaid.^33^ In addition, these states’ Medicaid claims were readily available to the study team given previous collaboration in the Pediatric Quality Measures Program.

Our study population consisted of youths ages 1 through 17 years with SCA who were enrolled in Medicaid for at least 1 continuous year within the study period. Continuous enrollment was required to ensure complete capture of claims; this approach reduced the likelihood of underestimating adherence rates due to missing data. For data through September 2015, youths with SCA were identified as those with at least 3 claims with an *International Classification of Diseases, Ninth Revision *(*ICD-9*) diagnosis code related to SCA (282.61 or 282.62). Compared with the criterion standard of newborn screening records, this case definition identified youths with SCA with a sensitivity of 91.4% and specificity of 80.0%. From October 2015 through December 2018, after the US transitioned to mandatory reporting using the *International Statistical Classification of Diseases and Related Health Problems, Tenth Revision *(*ICD-10*), youths with SCA were identified as those with at least 1 outpatient visit with an *ICD-10* diagnosis code of D5700, D5701, D5702, or D571. This case definition identified youths with SCA with a sensitivity of 94% and a specificity of 92% compared with newborn screening records.^34^ For each year of eligibility, we restricted our analyses to youths with no other forms of health insurance (eg, private insurance) to maximize the completeness of claims available. Records of youths were eligible to contribute data across multiple, nonsequential years.

### Outcome: Hydroxyurea Use

To identify filled prescriptions for hydroxyurea, a list of National Drug Codes was generated and verified using the RxNorm tool, which contains all medications available on the US market and is maintained by the National Library of Medicine.^35^ The number of days’ worth of supply of hydroxyurea was obtained for each filled prescription. Hydroxyurea use was characterized as the count of the annual days’ supply.^36^ Annual days’ supply was capped at 365 days.

### Statistical Analysis

All data were analyzed in SAS statistical software version 9.4 (SAS Institute). Each state analysis was run separately; given data use agreements, we could not share individual-level data across states. Levels of significance were set a priori at .05, and all tests were 2-sided. Frequencies and proportions were determined for demographic characteristics of the study population overall. Summary statistics of days’ supply of hydroxyurea were calculated by calendar year.

We conducted 2 analyses to assess changes in hydroxyurea use over time. First, we conducted a simple assessment of hydroxyurea use before and after guideline release. Guidelines were published in September 2014; therefore, we characterized 2010 to 2014 (2012-2014 for NYS) as the prerelease period and 2015 to 2018 as the postrelease period. The 2 measures of hydroxyurea use were collapsed within each time period and compared (before vs after release) using Wilcoxon rank sum tests.

Second, we assessed the entire study period for changes in rates of hydroxyurea use over time. A model for each state estimated days’ supply of filled hydroxyurea prescriptions. Prior to developing these models, we evaluated the distribution of zero days’ supply of filled hydroxyurea prescriptions in each state. In Michigan, the relative proportion of zeros was higher than the estimated number of zeros (ie, there was an excessive proportion of zeros for days’ supply relative to nonzero counts). In contrast, the proportion was less than 10% in NYS. Due to this more complex characterization of hydroxyurea, individuals with nonzero days’ supply were additionally modeled separately. Modeling approaches for each state are described subsequently. Finally, we conducted an additional analysis using the methodologies described previously but analyzing a different outcome: the count of hydroxyurea prescriptions filled within a calendar year irrespective of days’ supply.

#### Michigan

Michigan data were modeled with zero-inflated negative binomial regression to account for the number of person-years with zero days’ supply. Each model simultaneously assessed the probability of any hydroxyurea use (any days’ supply; the log-odds component of the binary classification of any vs none) and count of hydroxyurea use (number of days’ supply) measure (negative binomial component among individuals with nonzero days’ supply). For the log-odds component, covariates included year and age modeled linearly and an indicator for the pre– or post–guideline release period. The negative binomial component covariates included year modeled linearly, a quadratic term for year, an indicator for the pre– or post–guideline release period, and interactions between the before and after indicator and year variables.

#### New York

NYS data were modeled with negative binomial regression. In contrast to models for Michigan, these models did not require estimating zero-inflated models owing to a smaller proportion of individuals with zero filled prescriptions in NYS. Each model assessed the count of hydroxyurea use measure (number of days’ supply). Components included year modeled linearly, a quadratic term for year, an indicator for the pre– and post–guideline release period, and interactions between the before and after indicator and year variables.

Models used generalized estimating equations with robust standard errors to account for clustering of multiple observations per youth. The direction and significance of the year variable, as well as the interaction between time and the release indicator, were examined to understand trends over time for each outcome.

We performed a sensitivity analysis among a subset of youths with evidence of prior hydroxyurea use to assess the robustness of our results. Among these youths, we calculated descriptive statistics regarding days’ supply among youths who had at least 1 filled hydroxyurea prescription within the year. We then compared pre– and post–guideline release periods for each outcome using Wilcoxon rank sum tests.

## Results

In both states, a total of 4302 youths with SCA (2236 males [52.0%]; 2676 born 2005-2017 [62.2%]; 150 Hispanic [3.5%], 2929 non-Hispanic Black [68.0%], and 389 non-Hispanic White [9.0%]) contributed 12 565 person-years in the study. Across the study period, individuals in more recent birth years were represented in higher proportions; sex was equally represented (Table 1).

**Table 1.** Demographic Characteristics

Characteristic	Youths, No. (%) (N = 4302)	
	Michigan (n=885)	New York (n=3417)
Sex		
Male	459 (48.1)	1777 (52.0)
Female	426 (51.9)	1640 (48.0)
Race and ethnicity		
American Indian or Alaska Native, non-Hispanic	<5 (<1.0)	47 (1.4)
Asian or Pacific Islander, non-Hispanic	<5 (<1.0)	73 (2.1)
Black, non-Hispanic	762 (86.1)	2167 (63.4)
Hispanic, regardless of race	17 (1.9)	133 (3.9)
White, non-Hispanic	34 (3.8)	355 (10.4)
Unknown, non-Hispanic	70 (7.9)	642 (18.8)
Birth year		
1993-1996	104 (11.8)	151 (4.4)
1997-2000	133 (15.0)	458 (13.4)
2001-2004	147 (16.6)	633 (18.5)
2005-2008	177 (20.0)	665 (19.5)
2009-2012	187 (21.1)	778 (22.8)
2013-2017	137 (15.5)	732 (21.4)

### Michigan

A total of 885 youths (459 males [48.1%]; 501 born 2005-2017 [56.6%]; 17 Hispanic [1.9%], 762 non-Hispanic Black [86.1%], and 34 non-Hispanic White [3.8%]) ages 1 through 17 years were identified as having been enrolled in Michigan Medicaid for at least 1 year between 2010 and 2018, contributing 2914 person-years (Table 1). From 2010 to 2018, the mean (SD) annual days’ supply of hydroxyurea was 47.2 (93.6) days per child and ranged from 33.2 (78.0) days to 61.3 (107.4) days across study years, or a maximum of 16.8% of the year (Figure). Overall, most youths (2107 person-years [72.3%]) had zero days’ supply; among 807 person-years with non-zero days’ supply, the mean (SD) was 170.0 (103.5) days. There were 1990 person-years (68.3%) without a filled hydroxyurea prescription.

**Figure.**
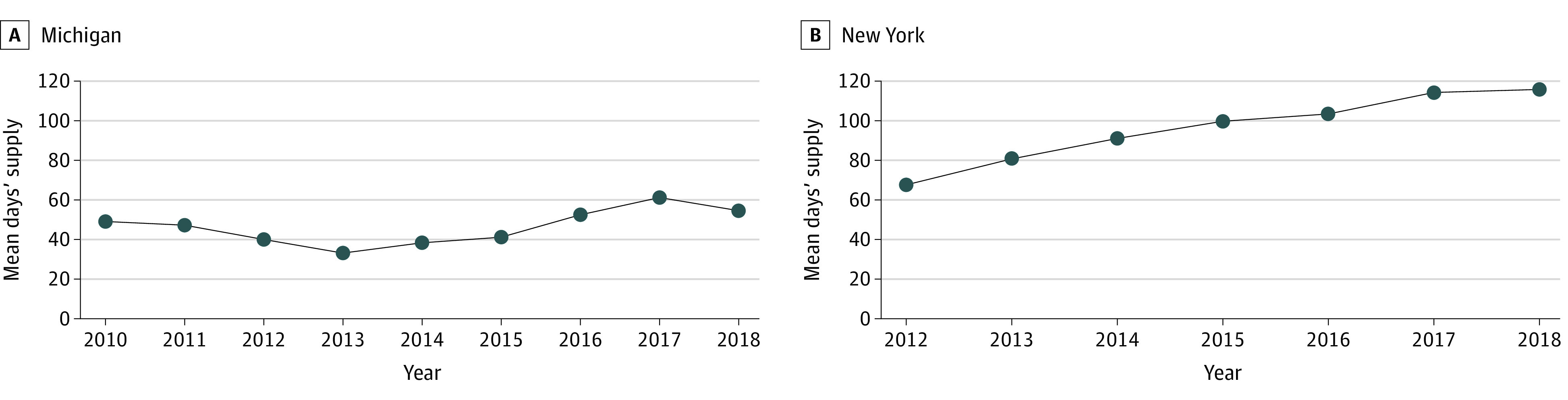
Mean Annual Days’ Supply of Hydroxyurea

The median (IQR) days’ supply for hydroxyurea was 0 (0-0) days before and 0 (0-60) days after release of the guidelines (Table 2). The before vs after guideline release comparison indicated that the mean (SD) annual days’ supply of hydroxyurea increased significantly, from 41.7 (89.9) days to 52.7 (97.0) days per child (*P* < .001) (Table 2). Before guideline release, 57 of 1472 person-years (3.9%) had 360 or more days’ supply of hydroxyurea; after guideline release, 75 of 1443 person-years (5.2%) had 360 or more days’ supply of hydroxyurea.

**Table 2.** Hydroxyurea Use Before and After New Treatment Guidelines

Measure	Days’ supply hydroxyurea, No. (N = 12 565 person-y)				
	Michigan (n = 2914 person-y)			New York (n = 9651 person-y)	
	Before release (n = 1472 person-y)^a^	After release (n= 1443 person-y)^a^	Before release (n = 3718 person-y)^a^		After release (n = 5933 person-y)^a^
Minimum	0	0	0		0
Quartile 1	0	0	0		0
Median	0	0	0		0
Quartile 3	0	60	148		240
Mean (SD)^b^	41.73 (89.88)	52.73 (97.04)	79.78 (125.32)		108.37 (142.77)

^a^ The prerelease period was 2010 to 2014 for Michigan and 2012 to 2014 for New York, and the postrelease period was 2015 to 2018.

^b^ Rankings were compared using Wilcoxon test. *P* < .001 for before vs after release in Michigan and New York.

The odds of having a nonzero days’ supply of hydroxyurea were 52% higher after release of the guidelines compared with the prerelease period (odds ratio, 1.52; 95% CI, 1.07 to 2.14). Among individuals with a nonzero days’ supply, there was an absolute decrease in the days’ supply after release of the guidelines (β after release = −3.66; 95% CI, −6.30 to −1.02). However, the slope of days’ supply decreased over the prerelease period (β for trend = −0.11; 95% CI, −0.24 to 0.03) but significantly increased in the postrelease period (β for trend = 0.94; 95% CI, 0.18 to 1.70) (Table 3).

**Table 3.** Regression Results Estimating Days’ Supply

Component	Estimated days’ supply (95% CI)	
	Michigan (n=2915 person-y)^a^	New York(n=4101 person-y)^b^
Logistic component (estimating any vs no hydroxyurea use measures)		
Years since 2010	0.00 (−0.07 to 0.06)	NA
Age	0.09 (0.07 to −0.11)	NA
Postguideline changes period^c^	0.42 (0.07 to 0.76)	NA
Negative binomial component (estimating count of hydroxyurea use measure)		
Years since 2010	−0.13 (−0.30 to 0.05)	0.20 (−0.26 to 0.66)
(Years since 2010)^2^	0.02 (−0.03 to 0.06)	−0.03 (−0.25 to 0.20)
Postguideline changes period^c^	−3.66 (−6.30 to −1.02)	0.11 (−1.53 to 1.75)
Years since 2010 × postguideline changes period^c^	1.14 (0.30 to 1.99)	−0.09 (−0.98 to 0.79)
(Years since 2010)^2^ × postguideline changes period^c^	−0.09 (−0.16 to −0.01)	0.02 (−0.22 to 0.26)

Abbreviation: NA, not applicable.

^a^ Michigan data were modeled with zero-inflated negative binomial regression.

^b^ New York State models include years since 2012. New York data were modeled with negative binomial regression.

^c^ Reference = preguideline changes period.

Among the subset of youths with SCA who had a nonzero days’ supply, the mean (SD) annual days’ supply of hydroxyurea was 170.0 (103.5) days. By year, the mean (SD) annual day’s supply ranged from 139.2 (100.5) days for 2015 to 196.5 (102.7) days for 2010 (eTable 1 in Supplement 1). Our before vs after guideline release assessment did not show a significant difference in either hydroxyurea use measure between time periods relative to guideline release (eTable 1 in Supplement 1). The analysis of counts of filled prescriptions showed similar results (eTable 2 and eTable 3 in Supplement 1).

### New York

A total of 3417 youths (1777 males [52.0%]; 2175 born 2005-2017 [63.7%]; 133 Hispanic [3.9%], 2167 non-Hispanic Black [63.4%], and 355 non-Hispanic White [10.4%]) ages 1 through 17 years with SCA were identified as having been enrolled in NYS Medicaid for at least 1 year between 2012 and 2018, contributing 9651 person-years (Table 1). From 2012 to 2018, the mean (SD) annual days’ supply of hydroxyurea was 97.4 (137.0) days, which increased from 67.6 (114.0) days to 115.8 (146.2) days across study years, or a maximum of 31.7% of the year (Figure). Overall, most youths (8252 person-years [85.5%]) had zero days’ supply; among youths with nonzero days’ supply, the mean (SD) was 229.1 (118.3) days. There were 8217 person-years (88.3%) without a filled hydroxyurea prescription.

The median (IQR) days’ supply for hydroxyurea was 0 (0-148) days for before and 0 (0-240) days for after release of the guidelines (Table 2). The before vs after guideline release comparison indicated that the mean (SD) annual days’ supply of hydroxyurea increased significantly from 79.8 (125.3) days to 108.4 (142.8) days (*P* < .001) (Table 2). The proportion of youths with at least 1 hydroxyurea fill increased from 418 of 1225 youths (34.1%) in measure year 2012 to 739 of 1521 youths (48.6%) in 2018. Preguideline release, 246 of 3718 person-years (6.6%) had 360 or more days’ supply of hydroxyurea; postguideline release, 831 of 5933 person-years (14.0%) had 360 or more days’ supply of hydroxyurea.

Among the subset of youths with SCA who had nonzero days’ supply within a year, the mean (SD) annual days’ supply of hydroxyurea was 229.1 (118.3) days. By year, the mean (SD) days’ supply ranged from 198.4 (110.2) days in 2012 to 243.9 (116.2) days in 2017; the mean (SD) annual number of filled prescriptions for hydroxyurea was 7.6 (4.2) prescriptions, ranging by year from 7.0 (4.1) prescriptions in 2012 to 7.8 (4.2) prescriptions in 2017 (eTable 1 in Supplement 1). Our before vs after guideline release assessment showed a significant increase between time periods (eTable 1 in Supplement 1). The analysis of counts of filled prescriptions showed similar results (eTable 2 and eTable 3 in Supplement 1).

## Discussion

Findings from this cross-sectional study suggest that few youths with SCA received the vital therapy provided by hydroxyurea despite national guidelines recommending its use. Specifically, 68.3% of person-years in Michigan and 88.3% of person-years in NYS with SCA did not have filled hydroxyurea prescriptions. However, we found encouraging trends in Michigan and NYS Medicaid programs suggesting modest increases in hydroxyurea use since release of the new guidelines. Given the effectiveness of hydroxyurea in reducing the frequency of disease-associated complications among this historically marginalized population, our findings underscore the importance of interventions to promote increased use of hydroxyurea.^18,19,20,21,22^

Prior to the release of the updated of the NHLBI guidelines, use of hydroxyurea was modest among youths with severe SCA; in 1 study, less than one-third of youths were adherent to hydroxyurea based on filled prescription claims when limited to those who had initiated hydroxyurea.^18,24,25,26,27^ In 2014, updated recommendations summarized the safety evidence and clarified the goal of encouraging greater patient use by reducing the minimum age and greatly widening the criteria for which people living with SCA should be offered hydroxyurea. Our analysis identified an increase in likelihood of youths initiating hydroxyurea after 2015 compared with prior years in Michigan. This trend was similar to those found in other studies from North Carolina and NYS suggesting that an increased proportion of youths with SCA had filled hydroxyurea prescriptions compared with previous time periods.^28,29^ Our study expands upon previous findings by focusing on youths with SCA specifically as opposed to the broader diagnosis of sickle cell disease,^28^ leveraging administrative claims instead of pharmacy data only,^30^ and performing an interrupted time-series analysis to evaluate the association of the guidelines with outcomes.

Although encouraging trends in initiation of hydroxyurea were found in Michigan after guideline release, the mean days’ supply initially decreased. Within the period of observation, this decrease was followed by a steeper increase in hydroxyurea initiation after the guidelines were released. Nonetheless, the mean days’ supply across Michigan and NYS during that period remained low, with possession of hydroxyurea covering no more than 32% of the year in the year with the highest level. Lower levels of possession, and therefore likely lower levels of medication use, are likely associated with reduced positive outcomes of this important medication among youths with SCA.^24,37,38,39^

Our findings suggest that interventions to increase uptake and ongoing use of disease-modifying therapies for SCA may be needed. This is not unique among chronic childhood and adolescence conditions given that medication uptake and consistent use for other conditions are often incomplete.^40,41,42,43^ Modestly scaled interventions have been associated with some improvement in hydroxyurea use within the context of clinical trials designed to address adherence issues. For example, Creary et al^44,45,46^ found that electronic directly observed therapy was feasible and acceptable and associated with high adherence to hydroxyurea use. Additionally, incorporating clinical pharmacists into the care team to support education and support for families has been shown to be associated with successful outcomes for other chronic conditions; this approach may be particularly well suited to hydroxyurea given that this medication requires significant dosage monitoring.^17,47,48^ However, an important consideration for all interventions is the association of state-level Medicaid policy with hydroxyurea use. Variation may exist across states, as well as within states, in the coverage of hydroxyurea, outpatient visits, and associated lab monitoring. Furthermore, this coverage may change over time, with varying levels of difficulty in accessing covered benefits. The importance of these policies cannot be overlooked, particularly when they may inhibit access to necessary preventive services, such as hydroxyurea. Wide-scale implementation trials have not yet been conducted to assess improved uptake and sustainability of outcomes. Interventions must also factor in wider systemic issues associated with underresourced communities, racism, and cultural differences between clinicians and patients that have disproportionately outcomes among individuals living with SCD.^49,50,51,52,53^

### Limitations

This study has several limitations. First, use of administrative claims to identify the study population excluded individuals who had not received any SCA-related care. However, we anticipate that this proportion would be modest across the study period given previous validation studies of methods used for case identification.^34^ Second, use of administrative data for filled hydroxyurea prescriptions did not account for gaps between prescribed but unfilled medications (ie, not charged to Medicaid). Given that those data do not validate the assumption that a filled prescription was fully taken by the intended individual, our results may overestimate the days’ supply that was taken by the individual. Third, additional factors may have been associated with trends in hydroxyurea prescribing practices during periods of interest, such as publication of the BABY HUG trial of young children.^23^ Fourth, our results capture data from 2 state Medicaid programs and may not reflect national trends or trends among youths who were privately insured or uninsured. Furthermore, these states may not be representative of the quality of care received by youths with SCA in the rest of the country. Nonetheless, approximately 70% to 90% of youths with SCA are covered by Medicaid at some point.^33^ Fifth, the case definition used to identify youths with SCA changed in the middle of the study period due to the transition from *ICD-9* to *ICD-10*, with a higher sensitivity and specificity in the *ICD-10* case definition. However, we expect this change to have minimal outcomes for our results given the low uptake of hydroxyurea across the entire study period.

## Conclusions

This cross-sectional study found that hydroxyurea use among youths with SCA varied substantially between 2 states based on comparing days’ supply of filled hydroxyurea prescriptions during 2 multiyear periods assessed. A modest improvement in uptake was associated with time periods after the 2014 release of expanded guidelines encouraging hydroxyurea’s broader use among youths with SCA. Our results further support the need to address the multidimensional barriers to obtaining this essential medication. Reducing these barriers may likely require multifaceted approaches that include interventions aimed at clinicians, pharmacists, and families.

## Notes

Supplement 1. **eTable 1.** Hydroxyurea Use Before and After Release of New Treatment Guidelines Among Youths With ≥1 Filled Hydroxyurea Prescription in the Year **eTable 2.** Hydroxyurea Use Before and After Release of New Treatment Guidelines **eTable 3.** Regression Results Estimating Filled Prescriptions

Supplement 2.
Data Sharing Statement
