# Supplementary material for: Changes in Hydroxyurea Use Among Youths Enrolled in Medicaid With Sickle Cell Anemia After 2014 Revision of Clinical Guidelines
Source: JAMA Netw Open. 2023 Mar 24;6(3):e234584. doi: 10.1001/jamanetworkopen.2023.4584 (PMC10313146; doi:10.1001/jamanetworkopen.2023.4584)
Supplement: Supplement 1. — eTable 1. Hydroxyurea Use Before and After Release of New Treatment Guidelines Among Youths With ≥1 Filled Hydroxyurea Prescription in the Year eTable 2. Hydroxyurea Use Before and After Release of New Treatment Guidelines eTable 3. Regression Results Estimating Filled Prescriptions [file jamanetwopen-e234584-s001.pdf]

Supplemental Online Content

Reeves SL, Peng HK, Wing JJ, et al. Changes in hydroxyurea use among youths enrolled in Medicaid with sickle cell anemia after 2014 revision of clinical guidelines. *JAMA Netw Open.* 2023;6(3):e234584. doi:10.1001/jamanetworkopen.2023.4584

eTable 1. Hydroxyurea Use Before and After Release of New Treatment Guidelines Among Youths With ≥1 Filled Hydroxyurea Prescription in the Year

eTable 2. Hydroxyurea Use Before and After Release of New Treatment Guidelines

eTable 3. Regression Results Estimating Filled Prescriptions

This supplemental material has been provided by the authors to give readers additional information about their work.

© 2023 Reeves SL et al. *JAMA Network Open.*

eTable 1. Hydroxyurea Use Before and After Release of New Treatment Guidelines Among Youths With ≥1 Filled Hydroxyurea Prescription in the Year

	Michigan			New York		
	Pre-Release (2010-2014)	Post-Release (2015-2018)	*p*-value	Pre-Release (2012-2014)	Post-Release (2015-2018)	*p*-value
	(n = 352)	(n = 457)		(n = 706)	(n = 692)	
**Days' Supply**						
Minimum	15	12	--	0	1	---
Quartile 1	89	81	--	114	130	---
Median	174	150	--	228	256	---
Quartile 3	254.5	249	--	324	365	---
Maximum	365	365	--	365	365	----
Mean (SD)	174.53 (103.01)	166.51 (103.89)	0.22	215.27 (114.92)	236.14 (119.35)	<0.001
**Filled Prescriptions**						
Minimum	0	1	--	1	1	---
Quartile 1	3	3	--	4	4	---
Median	6	5	--	8	8	---
Quartile 3	9	9	--	11	11	---
Maximum	15	16	--	27	27	---
Mean (SD)	6.08 (3.65)	5.79 (3.66)	0.25	7.29 (3.97)	7.69 (4.34)	<0.001

© 2023 Reeves SL et al. *JAMA Network Open.*

eTable 2. Hydroxyurea Use Before and After Release of New Treatment Guidelines

	Michigan			New York		
	Pre-Release (2010-2014)	Post-Release (2015-2018)	*p*-value[a]	Pre-Release (2012-2014)	Post-Release (2015-2018)	*p*-value
	(n = 1472)	(n = 1443)		(n = 3718)	(n = 5933)	
**Filled Prescriptions**						
Minimum	0	0	--	0	0	---
Quartile 1	0	0	--	0	0	---
Median	0	0	--	0	0	---
Quartile 3	0	2	--	5	7	---
Maximum	15	16	--	27	27	---
Mean (SD)	1.45 (3.15)	1.83 (3.39)	<0.001	2.70 (4.27)	3.53 (4.83)	<0.001

[a]Rankings were compared using Wilcoxon test

© 2023 Reeves SL et al. *JAMA Network Open.*

eTable 3. Regression Results Estimating Filled Prescriptions

	**Michigan**	**New York State**[a]
	Filled Prescriptions (n=2915)	Filled Prescriptions (n=4101)
	Estimate (95% CI)	Estimate (95% CI)
**Logistic Component (Predicting Any vs None of Hydroxyurea Use Measures)**		
Years since 2010	0.00 (-0.07 to 0.07)	N/A
Age	0.09 (0.07 to -0.11)	N/A
Post-guideline changes (ref=pre-guideline changes)	0.43 (0.08 to 0.79)	N/A
**Negative Binomial Component (Predicting Count of Hydroxyurea Use Measure)**		
Years since 2010	-0.13 (-0.31 to 0.06)	0.16 (-0.15 to 0.47)
(Years since 2010)^2	0.01 (-0.03 to 0.06)	-0.02 (-0.17 to 0.13)
Post-guideline changes (ref=pre-guideline changes)	-4.44 (-7.30 to -1.58)	0.16 (-0.92 to 1.24)
Years since 2010 * post-guideline changes (ref=pre-guideline changes)	1.38 (0.47 to 2.29)	-0.10 (-0.68 to 0.49)
(Years since 2010)^2 * post-guideline changes (ref=pre-guideline changes)	-0.11 (-0.19 to -0.02)	0.02 (-0.14 to 0.18)

© 2023 Reeves SL et al. *JAMA Network Open.*
