# Supplementary material for: Changes in Hydroxyurea Use Among Youths Enrolled in Medicaid With Sickle Cell Anemia After 2014 Revision of Clinical Guidelines
Source: JAMA Netw Open. 2023 Mar 24;6(3):e234584. doi: 10.1001/jamanetworkopen.2023.4584 (PMC10313146; doi:10.1001/jamanetworkopen.2023.4584)
Supplement: Supplement 2. — Data Sharing Statement [file jamanetwopen-e234584-s002.pdf]

Data Sharing Statement

Reeves. Changes in Hydroxyurea Use Among Youths Enrolled in Medicaid With Sickle Cell Anemia After 2014 Revision of Clinical Guidelines. *JAMA Netw Open*. Published online March 24, 2023. doi:10.1001/jamanetworkopen.2023.4584

Data
Data available: No

© 2023 Reeves SL et al. *JAMA Network Open*.
